# Sources and trends of trace elements and polycyclic aromatic hydrocarbons in a shallow lake in the Mediterranean area from sediment archives of the Anthropocene

**DOI:** 10.1007/s11356-022-22939-4

**Published:** 2022-09-20

**Authors:** Paola Gravina, Bartolomeo Sebastiani, Federica Bruschi, Chiara Petroselli, Beatrice Moroni, Roberta Selvaggi, Enzo Goretti, Matteo Pallottini, Alessandro Ludovisi, David Cappelletti

**Affiliations:** Department of Chemistry Biology and Biotechnology, University of Perugia, via Elce di Sotto 8, Perugia, 60123 Umbria Italy

**Keywords:** Shallow lake, Sediment cores, Geochemical baseline, Trace elements, PAHs, Pb isotopes

## Abstract

In this study, the anthropogenic contamination in Trasimeno lake (Central Italy) was investigated using three sediment cores spanning over the last 150 years (Anthropocene) to identify the primary sources of pollution and quantify the level of contaminant enrichment in the basin. First, based on the relative cumulative frequency and linear regression methods, we obtained a geochemical baseline for the lake using the deeper parts of the sediment cores. The geochemical baseline allowed us to determine the values of trace elements enrichment factors. On this knowledge, as a second result, we were able to reconstruct the natural sources and the anthropogenic impact on the lake with a biennial resolution. This goal has been obtained by combining different inorganic and organic chemical proxies such as trace elements, polycyclic aromatic hydrocarbons, and lead isotope ratios and exploiting both principal component and factor analysis to associate chemical proxies to human-driven contamination processes. Five different groups of elements have been identified, one of which is of natural origin and four of anthropogenic origin. In particular, it was possible to identify the times and impacts of the industrial activities during the Second World War, which dispersed heavy metals in sediments. Moreover, we found evidence of the recent human activities that have characterized the surroundings of the basin, such as Pb inputs related to the use of gasoline and the enrichment of certain elements generally used in agricultural activities (such as P, Cu, and Mn) due to the development of this sector in the last 40 years.

**Supplementary Information:**

The online version contains supplementary material available at 10.1007/s11356-022-22939-4.

## Introduction

Freshwater ecosystems such as lakes and rivers are critical to understanding the effects of the environmental change driven by human activities, which have been particularly relevant in the Anthropocene (Dubois et al. 2018). To assess the impact of human activities, an undisturbed reference, such as pristine natural background sediments, is necessary to reflect the situation of the individual lake on a local or regional scale. A background concentration is defined as the concentration of trace elements before industrialization, so its level should reflect natural processes uninfluenced by human activities (Tapia et al. 2012; Tylmann 2005). However, this reference is not easy to obtain because of the rapid population growth, industrialization, and urbanization processes over the past two centuries.

In the present work, we focus on Trasimeno, a shallow lake in Central Italy, which, similar to other basins in the Mediterranean area, has been severely impacted by human activities, which have altered its sensitive hydrological status since the beginning of the twentieth century and affected the ecosystem and biota (Goretti et al. 2016). Recently, exploiting high-resolution stratigraphic archives (Gravina et al. 2022), we characterized the three major hydrological regimes of the lake, focussing on the variations of precipitation and sedimentation processes of endogenic carbonates, i.e., calcite and calcium carbonate phosphate, relating the presence of these compounds to natural and human-driven processes. This study allowed us to identify the range of the sedimentary archives less impacted by anthropogenic processes.

Herein, we started from this piece of knowledge to define a geochemical baseline (Matschullat et al. 2000) which allowed us to identify and date the primary sources of pollution (Wang et al. 2019) affecting the Trasimeno lake and quantify the degree of contaminant enrichment (Wang et al. 2019).

Three short sediment cores have been exploited to this aim and characterized in terms of major and trace elements, polycyclic aromatic hydrocarbons, and lead isotope ratios. The multi-proxy approaches, with combination of different inorganic and organic indicators, and the treatment of data with multivariate statistical techniques, are the key to accurately reconstruct the events related to human activities that occurred in the area adjacent to the basin (Li et al. 2021). Studying the source of metals is quite complicated, because they are ubiquitous and are emitted both from natural and anthropic sources (Cearreta et al. 2000; Callender 2014; Ghadimi 2014). This is one reason why it is important to combine them with other proxies, such as lead isotope ratios. Lead isotope ratio analysis has proved a powerful tool for identifying the origin of lead. Townsend and Seen (2012), with many studies using sediments as the historical archive of Pb contamination (Odigie et al. 2014; Komárek et al. 2008), because Pb exhibits significant natural variation in the relative proportions of its isotopes. Along with lead, also PAHs are very source-specific and less sensitive to alteration and destruction than other forms of organic matter, thus remaining unchanged even after burial in the lake sedimentary archives (Elmquist et al. 2007). Due to these characteristics, the study of the processes of emission, transport and deposition of the PAHs is very widespread, especially given their strong toxicity and potential for human exposure (Du and Jing 2018).

The significant human impacts have been classified into three periods covering the Anthropocene and encompass artificial water level manipulation, industrial activities in the pre-second World War period, and more recent eutrophication related to agricultural practices.

## Materials and methods

### Study area, sampling and processing

The Trasimeno lake is a shallow and closed basin located in Central Italy. Despite its large extension (124 *km*^2^), which makes it the largest lake of Central and Southern Italy, this lake is very shallow, and its bathymetry is very smooth (Figure SM1 − *a*). In the last two centuries, the hydrometric trend of the basin passed through 3 distinct phases: (i) an old phase (OP) (from 1860 to 1900), during which the water level exceeded the overflow level (257.5 m a.s.l.), allowing the discharge of suspended materials to the outside; (ii) a middle phase (MP) (from 1900 to 1960), during which the water level remained below the overflow level and also suffered a substantial lowering, causing saturation of the components in the water column; (iii) a young phase (YP) (from 1960 to 2010), during which the water level rose but not above the overflow level (Figure SM1− *b*) (Gravina et al. 2022). The present study has characterized three sediment cores, C1, C2 and C3, with depth below ground level of 95, 102 and 50 cm and with sedimentation rates of 0.21, 0.20 and 0.16 *g* *cm*^− 2^ *year*^− 1^ respectively (Gaino et al. 2012). Each core was extruded and sectioned, in a series of 1-cm intervals for the top 30 cm and in 2-cm intervals for the lower remaining. Then the cores were dated, obtaining a biennial resolution (Table SM2), covering the 1860–2010 period (Gaino et al. 2012). This study has also characterized superficial sediments sampled during 2018 (Figure SM1 − *a* and table in Figure SM2). Finally a portion of each section/superficial sample has been acid digest or extracted in a proper solvent for the subsequent chemical analyses (details in the Supplementary Material - Section 1). All the details of the sampling site and sediment cores processing are reported in Gravina et al. (2022).

### Chemical analysis

Major (Al, Fe, Ti, V, P and Mn) and trace elements (Co, Ni, Pb, Cr, Cu, Zn) concentrations were determined by inductively coupled plasma optical emission spectrometry (ICP-OES, Ultima 2, HORIBA Scientific) equipped with an ultrasonic nebulizer (CETAC Technologies, U-5000AT). Analytical wavelengths are reported in Table SM1. Quality assurance was provided by determining the elemental concentrations for duplicate samples and one reference material (Certified Reference material SS-1-Contaminated Soil). The recovery of total metal concentration varied between 79 and 130% among the different analytes. The Limit of Detection (LOD) of the methods ranged from 0.01 to 2.95 ppm (Table SM2). PAHs have been analyzed using a gas chromatography-mass spectrometry with a triple-axis detector (HP7890A/5975CVL - Agilent Technologies, USA), equipped with a low bleed Select-PAH capillary column (Agilent J&W, CP 7461). To monitor the recovery method, a mix of 13 surrogate counterparts was used as standard (average recovery > 70*%*). The determination of ^208^Pb/^207^Pb and ^206^Pb/^207^Pb ratios was performed by an Agilent 8900 ICP-MS/MS (100 version, Agilent Technologies, Japan), operated in the MS/MS mode. The collision reaction cell was pressurized with a NH_3_/He 15 % mixture, used as a damping gas to lower the RSD. The instrumental mass bias was corrected with the standard sample bracketing method using the lead isotopic standard SRM 981 from NIST (Gaithersburg, MD, USA) (Vanhaecke et al. 2009; Bazzano and Grotti 2014; Bazzano et al. 2021; Bertinetti et al. 2022). The Supplementary Material (Section 1 - Chemical analysis) reports more details on the analytical methodologies.

A standard principal component analysis (PCA) and a factor analysis (FA) have been performed on the chemical dataset with R - Version 1.2.5033 using the packages *tidyverse* and *factoextra*.

### Geochemical baseline and enrichment factors

In this study, the geochemical baseline (GBL) was obtained using the bottom portions of the 3 sediment cores (approximately 25 cm), considering that these portions represent the least contaminated sediments due to their deposition before 1900. Then, two different GBLs were calculated using passive methods, i.e., the relative cumulative frequency technique (RCF) and the linear regression method (LRM).

The RCF technique is commonly used to obtain GBL (Matschullat et al. 2000; Teng et al. 2009) of an individual element and is based on different slopes of the relative cumulative frequency content fitting curves for the natural origin and abnormal concentration. A bend of the slope in the upper part of the curve (higher values) can be used to distinguish between anthropogenically non-influenced samples (low values) and anthropogenically influenced samples (high values). The baseline is obtained from the data below the first inflection value. The data in the cumulative frequency curve are tested with a linear regression method, until achievement of the condition of p < 0.05 and R^2^ > 0.9. Otherwise the maximum value is removed and the procedure is repeated until the two criteria are both met.

The LRM allows obtaining the regional GBL from deep sediments by plotting the element towards the normalizer (Selvaggi et al. 2020). According to the principle of normalization, the relative proportions of metals within materials from a particular region tend to be constant, even if the absolute metal concentration varies between crustal elements from one region to another. In the present study, normalization was done with Al or Fe according to the best correlation with the element considered. The samples lying beyond the confidence interval of 95% have been labeled as anthropogenically influenced. In the linear relationship between each element of concern and the reference elements (e.g., Al or Fe), data lying out of the 95% confidence band are eliminated, a new linear equation is built with the updated data set and, the process goes on until all the data are within the 95% confident band.

The local GBL was used in the present study to improve the assessment of anthropogenic impact through the enrichment factor (EF). The EFs are calculated as:

$$EF = \frac{\Big(\frac{[Me]}{[X]}\Big)_{sample}} {\Big(\frac{[Me]}{[X]} \Big)_{reference}}$$

where [Me] is the concentration of the element, [X] is the concentration of the reference element (Al, Fe, V, Li or Sc); at the numerator, there is the sample, while at the denominator there is the reference baseline (Schropp et al. 1990). Al was chosen as the reference for the present study.

## Results and discussions

### Geochemical baseline

The two methods describe were screened to identify the better GBL as representative of the maximum pristine state of the sampled Trasimeno sediments, in order to quantify the enrichment of the trace elements through the EF factors.

**Fig. 1:**
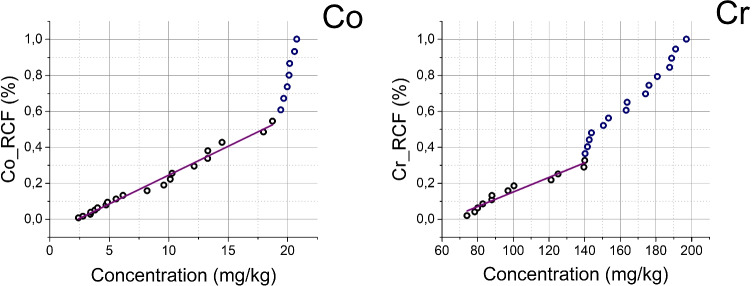
Cumulative frequency curves (scatters) of Co and Cr. Co represents the curve with only 1 inflection point, while Cr represents the curve with 2 inflection points. Linear regressions were performed on the cumulative frequency curve with p < 0.01 and R2 > 0.9 (purple line)

**Fig. 2:**
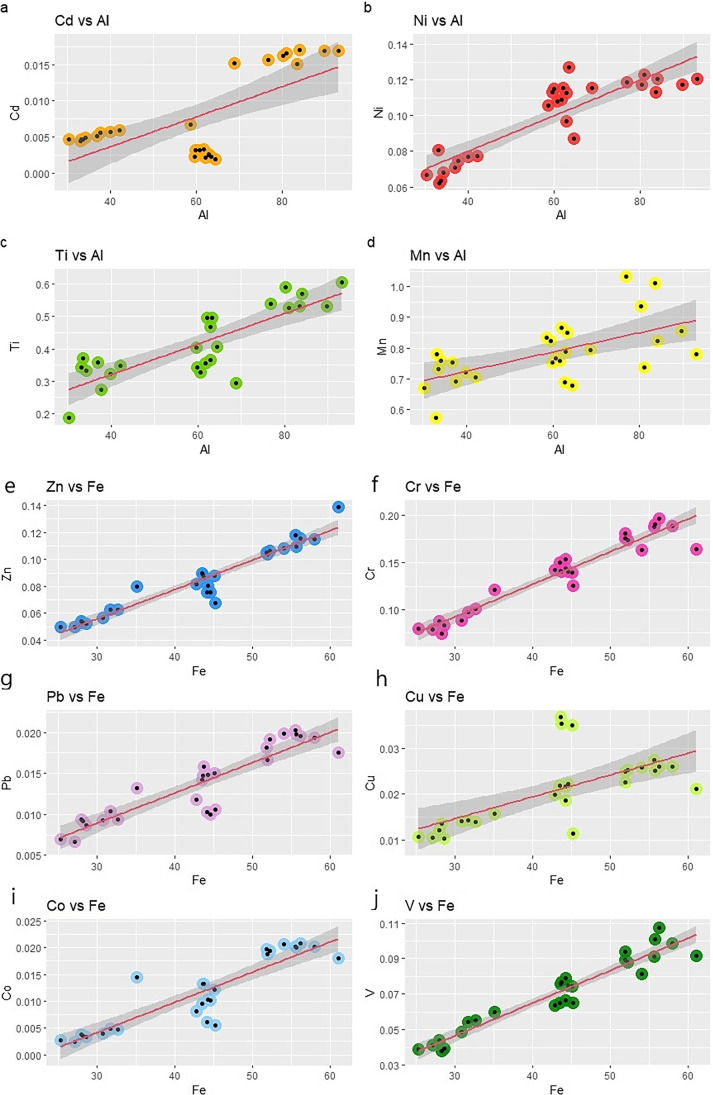
Normalization of heavy metals on the reference element: Al (from *a* to *d*) and Fe (from *e* to *j*) of bottom dataset. Concentration are in mg/kg. The linear regression was obtain with p < 0.01 and *R*^2^ greater. The dark gray area represents the 95% confidence

As an example of the RCF values provided by cumulative frequency curves method the cases of Co and Cr (other elements are in Supplementary Material in Figure SM4) are plotted in Fig. 1. Figure 2, instead, shows the linear regression method for calculating GBL, where Al or Fe are chosen according to the highest correlation value with the element considered. Both GBLs obtained through RCF and LRM are shown in Table 1. Eventually, Background (Bkg) values, also reported in Table 1, were calculated by arithmetic average of the bottom part of the cores (portion before the 1900s corresponding to the pre-industrial period).

**Table 1:** Concentration of geochemical baseline and background values calculated with different methods. All data are presented as mg/Kg or ppm

Methods	GBL / Bkg		Co	Ni	P	Pb	Cr	Cu	Ti	Zn	Mn	V
Relative cumulative	Core sed	**Mean**	8.46	72.82	1381.04	10.59	101.23	16.14	325.09	69.69	758.04	64.10
frequency methods (RCF)		dev sta	5.14	7.99	157.59	2.53	24.09	4.62	49.73	14.70	69.26	17.23
Linear regression	Core sed	**Mean**		85.83	1798.06				447.61		823.45	
technique — Al		dev sta		23.87	387.72				143.59		124.12	
Linear regression	Core sed	**Mean**	13.00			14.91	134.89	19.19		86.20		66.13
technique — Fe		dev sta	7.50			4.75	43.59	5.95		23.85		23.15
Baseline mean	Core sed	**Mean**	11.48	99.11	1680.05	13.73	137.42	20.82	415.41	84.19	784.26	70.48
3 bottom cores		dev sta	6.81	21.79	441.74	4.44	39.39	7.74	111.02	24.77	100.77	20.79
Bottom core C1	Core sed	**Mean**	10.66	108.98	1497.81	13.48	144.11	24.41	406.82	87.17	780.99	73.207
		dev sta	3.70	10.86	77.26	2.63	10.20	8.43	65.242	19.36	64.43	8.595
Bottom core C2	Core sed	**Mean**	19.93	118.13	2272.85	19.09	182.41	25.36	523.20	110.03	871.76	93.86
		dev sta	0.65	3.14	97.92	1.16	10.92	1.37	97.07	5.37	110.25	8.32
Bottom core C3	Core sed	**Mean**	4.87	71.23	1292.26	9.23	89.98	12.79	317.27	57.91	710.12	46.68
		dev sta	3.69	6.58	190.90	1.90	14.51	2.00	59.95	9.54	61.97	8.21
Current state	Core sed	**Mean**	10.7	76.6	418.6	18.8	100.0	18.2	292.5	78.5	1063.9	53.3
		dev sta	3.7	16.2	79.1	3.0	27.6	4.1	104.4	13.1	303.9	17.6
(Förstner and Wittmann 1981)	Lake sed	**Mean**		44.5		27	42	30		65.5		
Pre-indsutrial	Lake sed	**Mean**		40		22	48	34		97		
(Callender 2014)												
Recent lacustrine	Lake sed	**Mean**		39		102	63	60		207		
(Callender 2014)												

As shown in Table 1, the RCF is the closest lacustrine sediment baseline to other baseline values from different areas of the world (e.g., Callender 2014). As regards Ni, Cd and Cr the RCF baselines are higher than those reported by Callender (2014). While the RCF baselines for Pb, Cu and Zn are lower than Callender (2014) values, being 11, 16 and 70 ppm compared to 22, 34 and 97 ppm, respectively.

### Enrichment factors and trends of contaminants

EF values were calculated using the RCF baseline, considering that, even if widely used as a sediment metal-enrichment assessment tool, the calculated EF is highly dependent on the choice of an appropriate background or baseline as reference level (Rubio et al. 2000). Interpretation of EF values allows the assessment of contaminant enrichment. According to Birch et al. (1996), several scenarios can be depicted, i.e., EF < 1 “no enrichment”, 1 < EF < 3 “minor enrichment”, 3 < EF < 5 “moderately enrichment”, 5 < EF < 10 “moderately enrichment”, 10 < EF < 25 “severe enrichment” 25 < EF < 50 “very severe enrichment” and EF > 50 “extremely severe enrichment”. The EFs values were also interpreted by Zhang and Liu (2002): when EF is between 0.5 and 1, the metal could be mainly from the weathering process; while if EF is greater than 1.5, the metal is from anthropogenic sources or grater percentage of the metal is from non-natural process.

The boxplot, reported in Figure SM5 in Supplementary Material, shows means, high and low quartiles and outliers for the EF values in the 3 sediment cores (C1,C2,C3).

**Fig. 3:**
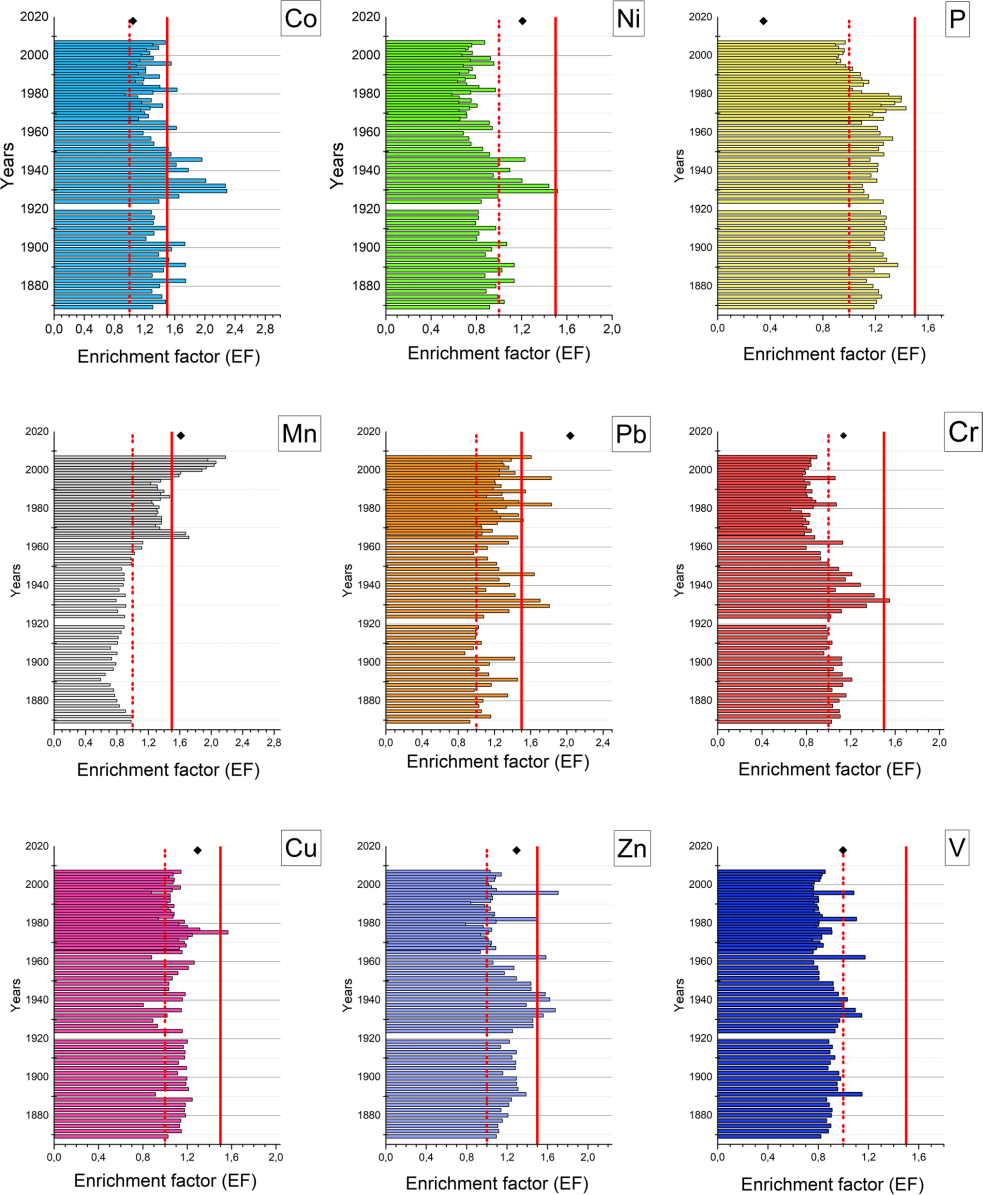
C2 core trend of enrichment factors for trace elements (Co, Ni, P, Mn, Pb, Cr, Cu, Zn and V) represented by the histogram graph. The black rumble represents the current state of enrichment in the surface sediments of the lake (corresponding to 2018). The dotted red line indicates limit at 1 (according to Birch et al. 1996), while continuous red line indicates limit at 1.5 (according to Zhang and Liu 2002)

The enriched elements in C2 (Fig. 3), listed in descending order, are Co, Pb, Zn, Mn, P, Cu, Ti, Cr, V, and Ni. The enriched elements, listed in descending order for C1 core, are Cu, Pb, Co, Ni, Zn, Cr, V, Mn, P, and Ti, while for C3 core are Pb, Zn, Cu, Ni, Cr, V, Mn, Co, Ti, and P. The global anthropogenic enrichment in the basin seems to be high, especially for Pb, Cu, Co, and Zn.

### Trends of polycyclic aromatic hydrocarbons

Specific organic proxies such as some polycyclic aromatic hydrocarbons (PAHs) allow to identify their possible source, which can be petrogenic, pyrogenic, biogenic, and diagenetic (Abdel-Shafy and Mansour 2016). The pyrogenic kind of PAHs is linked to exposure to high temperatures under low oxygen or no-oxygen conditions, such as wood and coal combustion. The petrogenic origin is due to transportation, storage, and spills of crude oil and crude oil products. In contrast, the biological origin of some PAHs is due to their synthesis by certain plants and bacteria or to their formation during degradation of vegetative matter. For the estimation of anthropogenic sources, Perylene is usually removed from the sum of PAHs, because it is formed after deposition transformation during diagenesis and is derived from natural precursors (Jiang et al. 2000). Therefore, the total PAH (=ΣPAHs) is calculated by subtracting the concentration of perylene (Fernández et al. 2002).

**Fig. 4:**
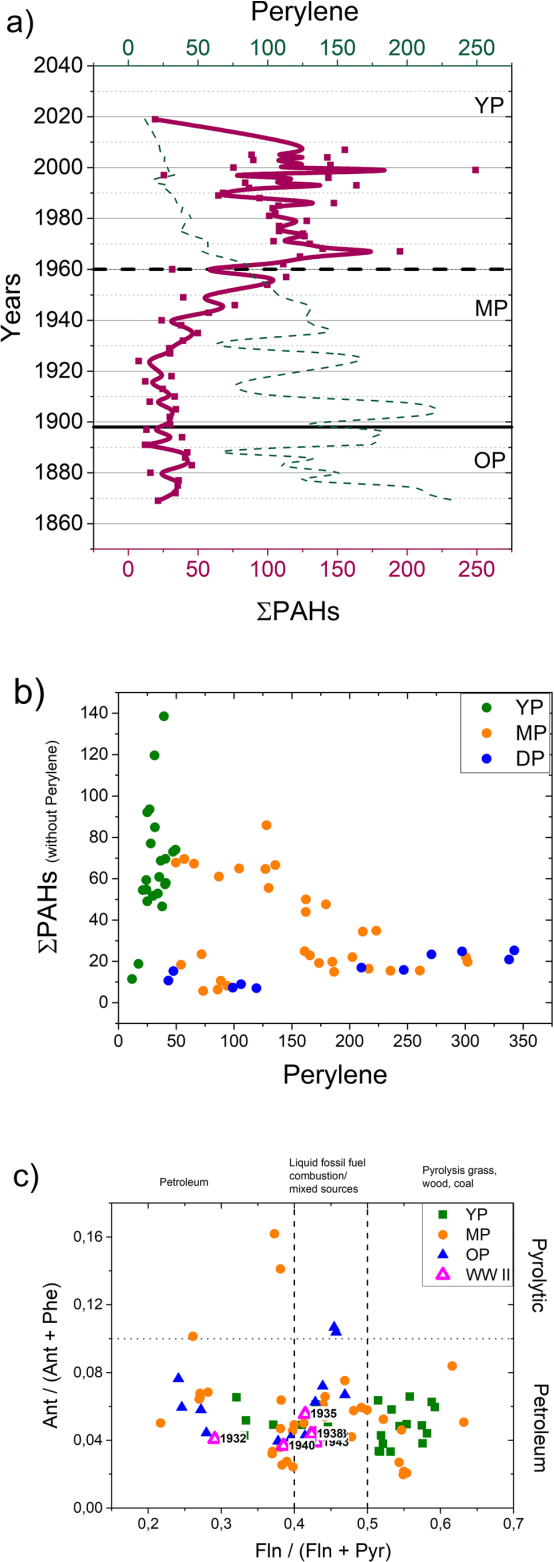
*a*) ΣPAHs (continuous red curve) and perylene (dashed green line) concentration (ng/g) in core C2; *b*) ΣPAHs versus perylene concentration (diagenetic origin) in core C2; *c*) scatterplot of (Fln/(Fln + Pyr) [Fln=Fluoranthene, Pry=Pyrene] and Ant/(Ant + Phe) [Ant=Anthracene, Phe=Phenanthrene] diagnostic ratios related to the core C2, with marked regions corresponding to different sources. Square represent samples in YP, circles represent sample in MP, triangles represent sample in OP and empty triangles represent samples during the 2nd World War (WWII)

As shown in Fig. 4a, ΣPAHs, measured on sediments in the center of the lake, core C2, was relatively constant until 1920 with an average value of 26.5 ng/g. This value is consistent with those found in surface sediments of relatively pristine environments in the Italian Alps (Poma et al. 2017). In the MP hydrological phase we observed a significant increase of ΣPAHs, starting in 1930–1940, and reaching values a factor 4 higher in the YP (Young phase), after WWII (Second World War) with a first maximum in 1965–1970 and a second maximum in the late 1990s (Fig. 4a).

The overall increase in the PAHs concentration found in the Trasimeno since 1930 has also been observed in other European lakes (Fernández et al. 2002; Du and Jing 2018). In detail, the first ramp of growth with the first maximum between 1960 and 1975 coincides with the exponential increase in the PAHs concentration found in the sediments of Swedish lakes, occurred between 1920 and 1960 (Elmquist et al. 2007). Since 1970, there was a break in the trend and a decrease in the PAHs concentration, reflecting the decreasing effects of dependence on petroleum fuels and the legislative actions aimed at reducing emissions. Finally, the second growth ramp with maximum concentration in the late 1990s, may be an indication of an increase in the consumption of wood pellets for domestic heating (Elmquist et al. 2007).

On the other side, Perylene remained constant and low during the most recent YP phase but increased rapidly with the depth of burial in sediments due to diagenesis, unlike other PAHs molecules (as can be seen from Fig. 4b). We exploited specific diagnostic ratios (typically based on isomers) to identify the contamination sources (Roszko et al. 2020; Yunker et al. 2002), assuming that different PAH molecules transform and degrade at the same rate during their lifetime in the environment, so that the characteristic concentration ratios of the sources are preserved. Compared to the many commonly used PAH diagnostic ratios (Du and Jing 2018), the scatterplot in Fig. 4c shows only two ratios ((Fln/(Fln + Pyr) and Ant/(Ant + Phe)) that were the most significant for this case study. In detail, the Fln/(Fln + Pyr) ratio distinguishes the origin of samples belonging to different historical periods (Guo et al. 2011). The Trasimeno sediment samples from the period of most significant industrial activity, i.e., around the Second World War, indicate oil and liquid fossil fuel combustion sources. Most YP samples represented anthropogenic origin related to biomass combustion and generated by pyrolysis, which can be associated with characteristic agricultural activities of the surroundings of Trasimeno lake in the last part of the twentieth century.

### Identification of anthropogenic sources by PCA and FA analysis

**Fig. 5:**
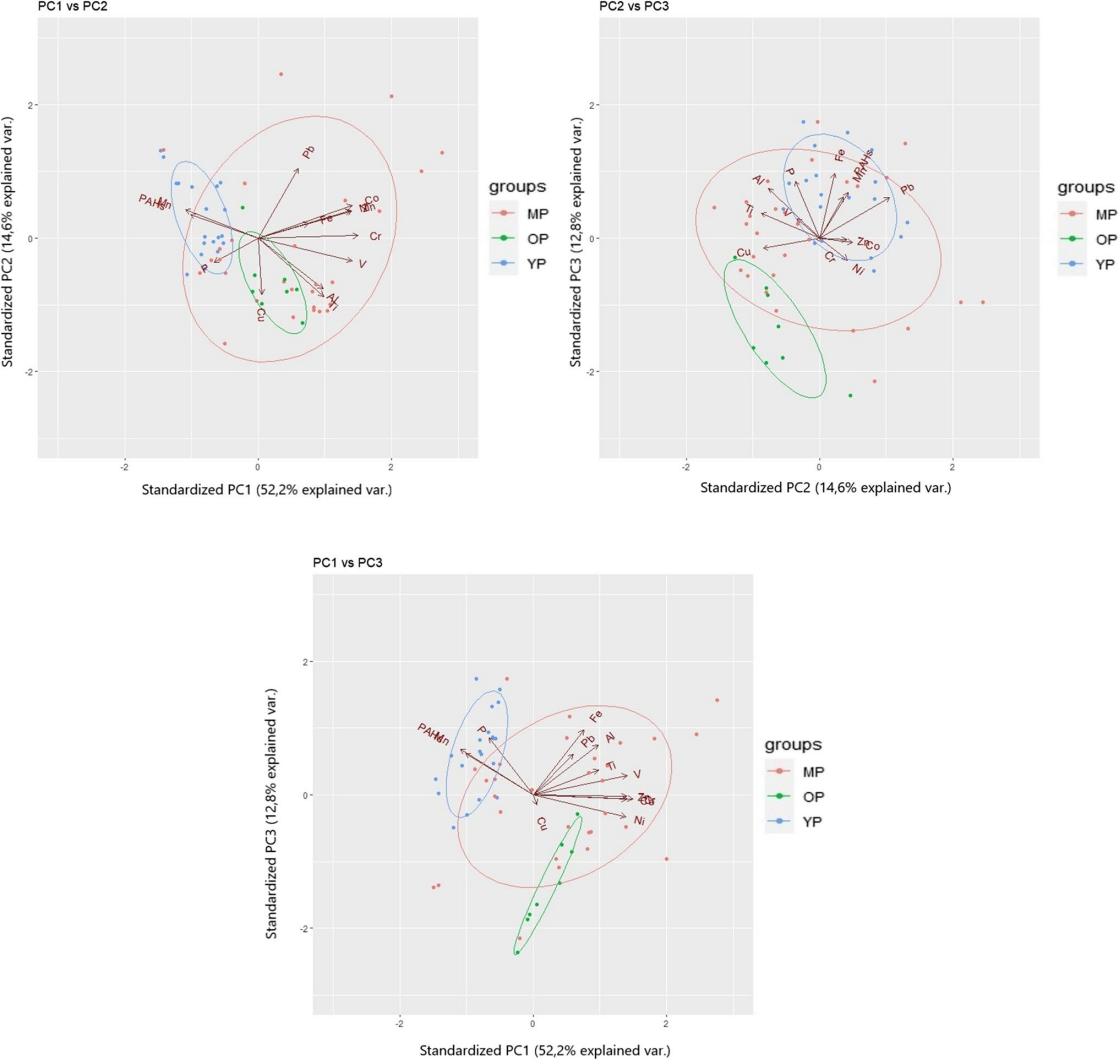
C2 core PCA: the different major principal components against each other are reported in each graph plot, comparing trace elements, heavy metals, phosphorus and ΣPAHs. The legend shows the scores according to specific hydrometric phase: phase OP in green, phase MP in red and phase YP in blue

A PCA analysis was performed on the C2 central core, which has the largest number of samples and which is more undisturbed and representative of the entire basin. The PCA performed on the C2 included trace elements and ΣPAHs (Fig. 5 and Table SM4). The analysis, especially the PC2 component, identified 5 groups of tracers: one natural group (A) including Al, Ti, V and Fe and 4 distinct anthropogenic groups (B, C; D and Pb). Group *B* included Co, Ni, Zn and Cr, group *C* included Cu and P, and *D* included Mn and ΣPAHs. In addition, the Mn variable is explained in part also by PC1. Pb is an isolated element that is separated from the groups mentioned above and is explained mainly by PC2.

The score legend of Fig. 5 effectively separates the three different hydrometric phases that have occurred in the lake over the past 150 years (OP, MP and YP), and allows to determine the sources of elements and organic compounds. In particular, the YP scores and PC2 distinguished the anthropogenic element groups (B, C and D groups). The distinct groups of anthropogenic elements have different origins; in fact, the anthropogenic group *B* is well separated from the natural elements (groups *A*) by PC2, but is also well isolated from Pb, which is likely associated with atmospheric deposition due to human use of leaded gasoline since the 1960s.

Finally, the results of the PCA analysis individuated a specific type of contamination throughout the lake, influenced by the intensification of agriculture during the eutrophic phase (YP, after 1970). The use of fertilizers, which can contain Mn, Cu, P, Zn, and Cr, Pb, is the main impacting factor. Manganese can be emitted as Mn sulfates in fertilizers or from pesticides, animal feed, or water treatment products. On the other hand, copper is contained in pesticides and fungicides (used to control plant diseases) or is emitted as Cu sulfate (treatment used to control algal growth). Actually, Cu sulfate is also added to pig feed to suppress bacterial action and therefore it is poured in the soil when pig slurry is used as fertilizer (Panagos et al. 2018). The use of fungicides based on the mixture of Cu sulfate, lime and water started in the 1880s and became widely used to control downy mildew in grapevines, while on the global scale, the use of Cu in agriculture has increased since 1980 (Panagos et al. 2018). Ultimately, the use of both Cu and P as fertilizers has been a common practice for much of the last century (confirming their appearance in group *C*), in contrast, the use of Mn as a fertilizer was not approved in Europe until the early 2000s, and that may be the reason for Mn does not belong to group *C* (EFSA Panel (NDA) 2013; (FEEDAP) E 2016; Christensen et al. 2015). Mn and ΣPAHs growth trends are also very similar, showing strong increase in concentration especially in recent years. For this reason both belong to group *D*. However, no particular common source for Mn and ΣPAHs has been identified, mainly because it is difficult to attribute to such a complex parameter as ΣPAHs, a single source. Factor analysis (in Supplementary Material Figure SM7) confirms the source-dependent separation of elements obtained using the PCA. It is observed that Al and Fe are separated from other elements, representing the group of natural origin, while among the elements of anthropogenic origin, the PAHs are separated by Mn, while there is the subgroup Mn-P

### Industrial contamination in the middle hydrological phase

The average concentration of some major and trace elements (V, Co, Ni, P, Pb, Cr, Cu, Ti, Mn and Zn) measured for the three sediment cores (top part, bottom part — used for GBL calculation — and whole core) together with maximum and minimum values are reported in Supplementary Material in Table SM3. The trends are qualitatively similar for the three cores (Figure SM3). Nevertheless the highest concentrations have been recorded in C2 (center of the lake) and the lowest in C3 (Southern lake). These differences can be associated with the different sedimentation rates (Gaino et al. 2012), the distance from the shore or the center of the basin, and the different composition of washed-out material from the Eastern, Western, and Southwestern parts (Figure SM1) of the area surrounding the lake (Yang and Rose 2005). It results from Table SM3 that in the C2 core, Ni, Cr and Ti have higher concentrations in the bottom with respect to the top of the core, and the current (2018) concentration values drop even further. On the other side V, Co, Cu, Zn have comparable values along with the sediment core, but also show lower values in the most superficial and current sample; unlike previous trends, Pb and Mn present lower mean concentrations in the bottom part of the core with respect to the top part and also with respect to the current (2018) conditions. The situation is slightly different for the north-eastern C1 core: Cu, Pb, Co, Mn and Zn show lower mean concentration values in the bottom part than in the top part of the core and whole core while V presents stable values for the whole core record. In the case of the southern C3 core, V, Co, Ni, Pb, Cr, Cu and Zn present lower mean concentration values in the bottom part of the core than in both the top and the whole sediment core; Ti and Mn present relatively stable values for the entire core record (Table SM3 and Figure SM3).

**Fig. 6:**
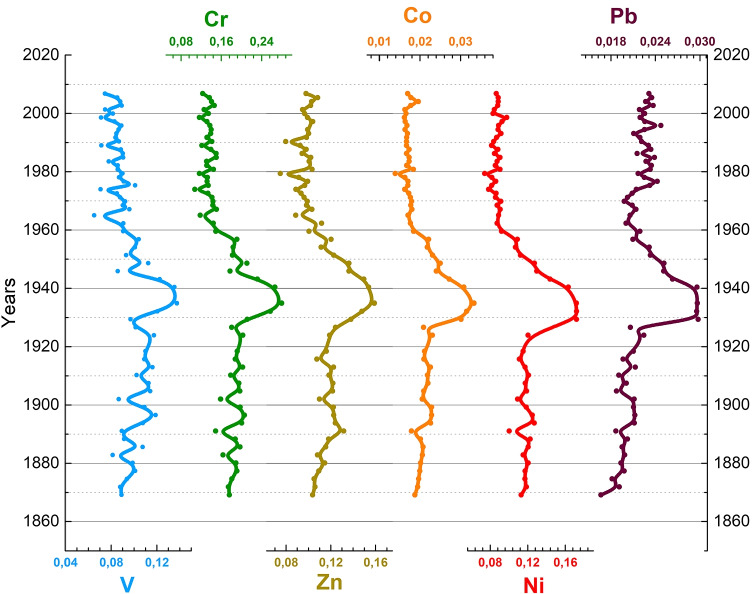
Trace metal concentration (V, Cr, Zn, Co, Ni, Pb) temporal profiles of the C2 core (adjacent-averaging method, with weighted average options was used on 5 points to obtain the trends line)

A closer view of the concentration profiles of some specific trace elements (Pb, Ni, Co, Zn, Cr, and V) of the C2 core is shown in Fig. 6. The concentration trends are characterized by a clear peak dated between 1930 and 1945 for all the elements. The peak corresponded to a simultaneous increase of the element concentrations and coincided with a relative rise in the water level (see Figure SM1 - *b*), though in the general context of the lowering phase. Overall, these pieces of evidence point to a significant contamination process lasting more or less 20 years. The nature of the trace elements involved suggests an industrial source. Indeed, in this period, a military airplane industry was operative and very active in Passignano, the main village on the lake’s northern coast. This airplane industry moved from Milan to the surroundings of the Trasimeno’s shores in 1916, and one of the most important Italian aeronautical schools was also established close to the basin. Technological development began in 1922 when the *“Societa*’*AeronauticaItaliana”* (*SAI*) was created and the combat aircraft production increased significantly with the outbreak of World War II (Bellaveglia 2015; SAI 2018).

**Fig. 7:**
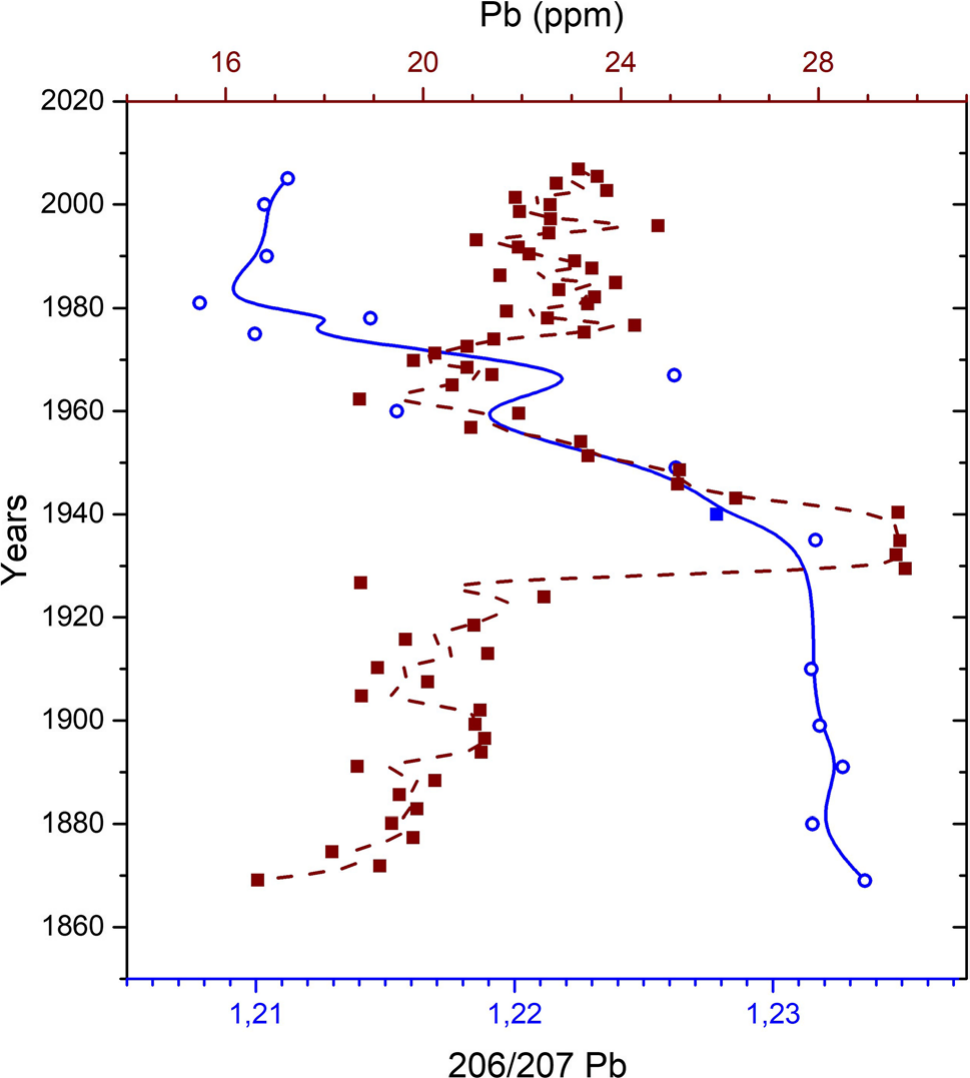
Trends of total Pb concentration and ^206^Pb/^207^Pb isotope ratio, for the C2 core. The solid blue line indicates the 206/207 Pb isotope ratio, while the dashed brown line indicates the Pb concentration (ppm), and adjacent-averaging method, with weighted average options was used on 5 points to obtain the trends line

After the peak, most elements lowered in concentration while, in the case of Pb, we notice a growth from the 1960s to the present day. This trend must be associated with a different source and reasonably connected with the use of leaded gasoline, containing Pb as an anti-knockout substance (Resongles et al. 2021). A further investigation for the Pb case has been made by measuring the ^208^Pb/^207^Pb and ^206^Pb/^207^Pb isotope ratios which are proxies of the emission source. Results are shown in Fig. 7 for the ^206^Pb/^207^Pb together with the trend of the total Pb concentration. A ^208^Pb/^207^Pb versus ^206^Pb/^207^Pb plot is presented in the Supplementary Material (Figure SM8). The results in Fig. 7 show a clear lowering of the ^206^Pb/^207^Pb isotope ratio from values larger than 1,230 (1,233 the maximum) before 1920, which can be considered a natural situation, towards values lower than 1,210 (1,207 the minimum) in the most recent years. Isotope ratio values can vary easily between different areas depending on the mineralogy of the area and on the level and type of contamination from the surrounding. In general, the values obtained in the Trasimeno lake are consistent with the literature (Odigie et al. 2014; Gobeil et al. 1995; Townsend and Seen 2012; Chiaradia et al. 1997; Bränvall et al. 2001). The lowering of the ^206^Pb/^207^Pb ratio plotted in Fig. 7 started clearly in coincidence with the pollution peak of the 1930–1940 and carried on further with a minimum in the 1980s and a slight recover later on. According to the literature this particular ratio is higher in natural soils, while it decreases because of anthropogenic pollution (Renberg et al. 2002; Sakata et al. 2018; Chiaradia et al. 1997; Bränvall et al. 2001). Moreover, a three-isotope plot can often help identifying and differentiating between anthropogenic and natural/geogenic Pb sources, particularly when the naturally occurring and anthropogenically introduced Pb has significantly different isotope ratios (Townsend and Seen 2012). We reported in the supplementary material (Figure SM8) the correlation between the ^208^Pb/^207^Pb and the ^206^Pb/^207^Pb isotope ratios which, consistently, suggest the contribution of at least two end-members to the observed Pb isotope ratio. In conclusion, the isotope ratio results confirm the anthropogenic nature of the contamination in the 1930–1940 event and the successive anthropogenic origin of Pb in the more recent years.

### Impacts of vehicular traffic and agricultural contaminations in the young hydrological phase

Pb shows a characteristic enrichment trend, which is in good agreement between the different sampling sites. In particular, the trend shows an increase in concentration during the WWII period that persisted until the early 2000s. As discussed above, Pb enrichment is primarily due to anthropogenic activities such as the use of leaded gasoline and the disposal of sewage sludge to the ground: only core C1 showed a lowering of enrichment after 2000, indicating the termination of leaded gasoline use, which came into effect in 2002 in Europe. The C1 core is located in the area of the lake most impacted by vehicular traffic due to the presence of a highway, built in the 1965, 200m far from the shore.

The intensification of the agricultural activities in the YP is responsible for the release of various elements and pollutants in the environment, such as the essential elements (Cu and Zn) contained in fertilizers, and the heavy metals (Pb and Cr) used in the synthesis of fertilizers as catalysts (Nacke et al. 2013). Moreover, the use of leaded fuel in agricultural machinery for the development of farming operations is another reason for enrichment of Pb (Nicholson et al. 2003; Zan et al. 2012).

Cu is consistently enriched in the sampled sediments, with maximum values recorded between 1970 and 1980. One of the main sources of Cu in the Trasimeno basin is the use of Cu sulfate pentahydrate in the production of fungicides, introduced in agriculture as early as the nineteenth century and widely distributed on fruit plants (olives and vines) and vegetables. Other sources of land pollution are sewage sludge, municipal compost and animal waste (Callender 2014). Mn enrichment began only later in 1965, with a maximum enrichment around the 1990s, confirming the delay in using this element for agricultural purposes towards the end of the century.

The current state of the sediment showed that pollution caused by some elements and heavy metals, such as Co, Ni , Cr, Zn, P and V has returned to baseline levels after increasing during the industrialization, urbanization and agricultural intensification. On the other hand, elements like Pb, Mn, Cu and Zn maintain enriched values relative to the baseline but lower than the period between 1940 and 1990 (Fig. 3). Mn and Cu are currently used in the predominant agricultural activities around the Trasimeno lake. The Pb enrichment may be due to the persistence of lead contamination even after the cessation of leaded petrol use in the early 2000s (Resongles et al. 2021). The process of remobilization of historical Pb deposited in soil from the atmosphere can be considered a critical current secondary source (MacKinnon et al. 2011).

## Conclusion

The main proxies of the Trasimeno lake’s pollution are industrial metals (Cr and Zn) and agriculture-related elements (Cu, Mn and P). The impact of industrial pollution at the regional level started before World War II, mainly tied up to the increase in the airplane industry activities, as demonstrated by Pb isotope ratios trend. Over time, the main pollution inputs to the Trasimeno lake shifted towards the intensification of agriculture, livestock, urbanization and transportation, which have increased since the 1960s. PAHs concentration and relative abundance, which is similar to that of other European lakes in the same period, helped to disentangle the effects of biomass and fossil fuels combustion processes.

The Trasimeno’s GBL values determined in this study are comparable with global baseline references from the literature, and they are also reasonable with respect to human activities around the lake, therefore ensuring a correct quantification of sediment enrichment by pollutants.

The assessment of the current condition of the lake demonstrated that some elements such as Co, Ni, Cr and V, mainly related with industrialization, urbanization and agriculture, have returned to GBL levels. On the other hand, Mn, Pb and Cu, coming from intensive agricultural activities are highly persistent in the environment even after being banned at the end of the last century, and remain enriched compared to GBL values.

## Supplementary Information

Below is the link to the electronic supplementary material.

Supplementary file1 (PDF 12.4 MB)

## Data Availability

Yes
