# Supplementary material for: Sources and trends of trace elements and polycyclic aromatic hydrocarbons in a shallow lake in the Mediterranean area from sediment archives of the Anthropocene
Source: Environ Sci Pollut Res Int. 2022 Sep 20;29(57):85815–28. doi: 10.1007/s11356-022-22939-4 (PMC9668802; doi:10.1007/s11356-022-22939-4)
Supplement: Supplementary file 1 — Supplementary file1 (PDF 12.4 MB) [file 11356_2022_22939_MOESM1_ESM.pdf]

# Sources and trends of trace elements and polycyclic aromatic hydrocarbons in a shallow lake in the Mediterranean area from sediment archives of the Anthropocene

Paola Gravina[1*], Bartolomeo Sebastiani[1], Federica Bruschi[1], Chiara Petroselli[1], Beatrice Moroni[1], Roberta Selvaggi[1], Enzo Goretti[1], Matteo Pallottini[1], Alessandro Ludovisi[1] and David Cappelletti[1]

[1*]Department of Chemistry Biology and Biotechnology, University of Perugia, via Elce di Sotto 8, Perugia, 60123, Umbria, Italy.

*Corresponding author(s). E-mail(s): paolagravi@gmail.com; Contributing authors: bartolomeo.sebastiani@unipg.it; federica.bruschi@studenti.unipg.it; petrosellichiara@gmail.com; b.moroni@tiscali.it; roberta.selvaggi@unipg.it; enzo.goretti@unipg.it; matteo.pallottini@unipg.it; alessandro.ludovisi@unipg.it; david.cappelletti@unipg.it;

## SUPPLEMENTARY MATERIAL

# 1 Chemical analysis

## 1.1 Major and trace elements

Major and trace elements (Al, Fe, Ti, V, Mn, Co, Ni, Pb, Cr, Cu, Zn and P, and corresponding wavelengths in Table SM1) concentrations were determined by Inductively Coupled Plasma an Optical Emission Spectrometry (ICP-OES, Ultima 2, HORIBA Scientific) equipped with an ultrasonic nebulizer (CETAC Technologies, U-5000AT). The sediment samples were first dried at 105 °C for 24 h, then an aliquot (0.250 g) was digested with 8 ml of ultrapure $HNO_3$ (65-69%) and 2 ml of ultrapure $H_2O_2$ (30-32%). The acid digestion was performed in a Mars Microwave Oven, with 2 working steps: the first at 130 °C (200 psi) for 1' and the second at 180 °C (300 psi) for 10'. The digestion mixture was finally filtered using 2,5 $\mu$m filters (Whatman Grade n $\hat{4}$2), and diluted to 50 ml with ultrapure $H_2O$ (18 M$\Omega$). Quality assurance was provided by determining the elemental concentrations for duplicate samples and one reference material (Certified Reference material SS-1-Contaminated Soil). The recovery of total metal concentration varied between 79 and 130% among the different analytes. The Limit of Detection (LOD) of the methods ranged from 0.01 to 2.95 ppm (Table SM2).

## 1.2 Organic compounds

An amount between about 2 to 4g dry weight of powdered sediment was spiked before extraction with a mix of 13 perdeuterated surrogate homologues as recovery standards (namely naphthalene-d8, acenaphthene-d8, phenanthrene-d10, fluoranthene-d10, pyrene-d10, chrysene-d12, benzo(b)fluoranthene-d12, Benzo(a)pyrene-d12, perylene-d12, debenzo(a,h)anthracene-d12, benzo(ghi)perylene-d12 and dibenzo(a,i)pyrene-d14, to monitor recovery method (average recovery >70%). The PAH fractions

were extracted twice in dichloromethane (DCM) by ultrasonic agitation at ambient temperature for 15min. Then, the combined extracts were fractionated by adsorption chromatography on activated silica gel (high-purity grade, pore size 60 , 70-230 mesh ASTM, Merck, Germany), pre-conditioned overnight at 120°C. The PAHs were isolated by elution with 20ml of a n-hexane-dichloromethane solution (4:1 ; v/v) and reduced to small volume. The residues was resuspended in 80 $\mu$l for GC-MS analysis. An aliquot of 0.5 $\mu$l in n-hexane was analysed using a gas chromatography-mass spectrometry with a triple-axis detector (HP7890A/5975CVL - Agilent Technologies, USA), equipped with low bleed Select-PAH capillary column (Agilent J&W, CP 7461), 15mx0,15mm ID and 0,10µm film thickness.+ 3m EZ-Guard.

GC oven programmed conditions were: from 80° (kept 0.8 min.) to 180°C at 70°C/min., to 230°C at 7°C/min. (kept 7 min.), to 280°C at 50°C/min. (kept 7 min.) and to 350°C at 30°C/min. (kept 7 min.) High purity Helium was used as carrier gas at constant flow rate of 1.2 ml/min and the injector operated under pulsed splitless mode (50psi for 0.4 min.) at 300°C. The mass detection was performed in selected ion monitoring (SIM) mode with ion source operating in electron positive ionisation at a voltage of 70eV and temperature of 230°C. All the reagents used for the analytical procedure, extraction and clean-up were analytical grade of highest purity or high performance liquid chromatography (HPLC) grade ($\geq$99%) and purchased from the Sigma-Aldrich, Deisenhofen, Germany. The analytical standard of PAHs and their perdeuterated surrogate homologues were supplied by BCR Certified Reference Material CEE, Lab Service Analytica-Dr. Ehrenstorfer GmbH (Italy-Germany) and Neochema GmbH (Germany). The procedure was defined according to ISTISAN 99/28 (Italian

National Health Service, 1999), an extensively consolidated method for quality assurance and quality control (QA/QC) in analysis of Persistent Organic Pollutants (POPs) in sediments.

## 1.3 Lead isotope analysis

The determination of $^{208}Pb/^{207}Pb$ and $^{206}Pb/^{207}Pb$ ratios was performed by an Agilent 8900 ICP-MS/MS (100 version, Agilent Technologies, Japan), operated in the MS/MS mode. The instrument is equipped with two quadrupole mass units (Q1 and Q2) and a third generation octopole-based collision/reaction cell system (CRS) located in-between (Q1-CRC-Q2). A micro mist nebulizer and a spray chamber kept at 4°C were used for the sample introduction. The CRC was pressurized with a $NH_3$/He 15 % mixture, used as a damping gas to lower the RSD. Q1 and Q2 were both set on mass $m/z$ = 206, 207 and 208. The integration time for each isotope mass was set to 1 second with 4000 sweeps. Internal precision was 0.56% (%RSD, n = 10) for the $^{206}Pb/^{207}Pb$ ratio. The instrumental mass bias was corrected with the standard sample bracketing method using the lead isotopic standard SRM 981 from NIST (Gaithersburg, MD, USA) Vanhaecke et al (2009). More details on the method are reported in Bazzano and Grotti (2014); Bazzano et al (2021); Bertinetti et al (2022)).

**Acknowledgments.** We thank MIUR and University of Perugia for financial support through AMIS project ("Dipartimenti di Eccellenza–2018–2022").

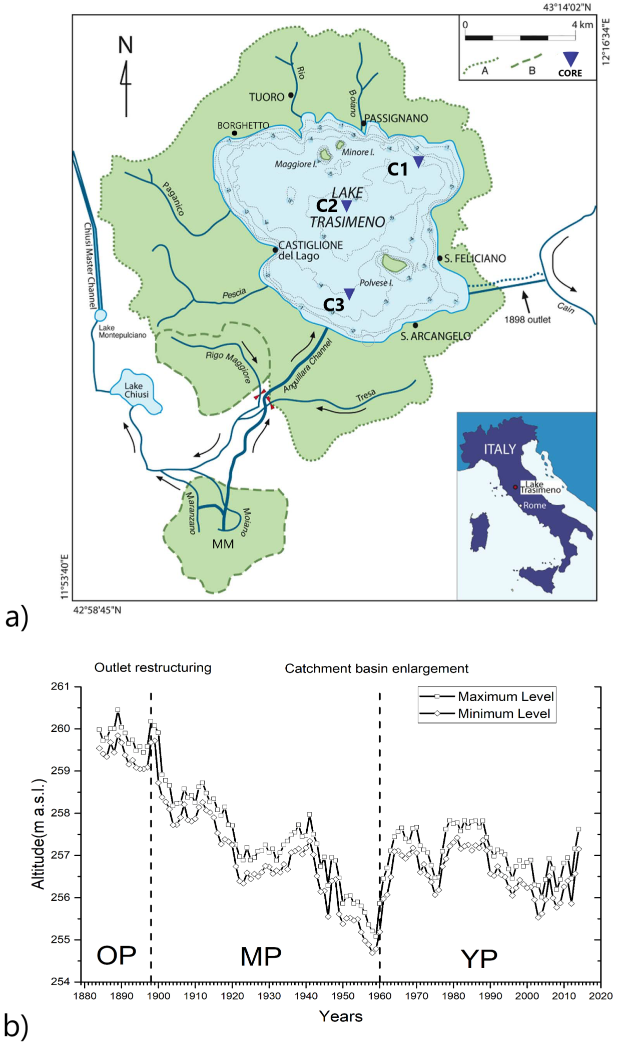


Fig. SM1: *a*) map of the Trasimeno lake with its catchment basin (modified from Marchegiano et al (2018)), A) natural catchment area, B) artificially-joined basins of Rigo Maggiore (RM) and Moiano and Maranzano (MM, is joined to the lake by artificial canal crossing over the natural waterway). The blue triangles mark the location of sampling sites (C1,C2 and C3 cores located at the coordinates N 43°09'40.0" E 12°10'06.0", N 43°08'38" E 12°06'43" and N 43°05'51" E 12°06'38", respectively). *b* shows the trend of water level, with hydrometric phases, old (OP), middle (MP) and young (YP) are indicated.

| Sample code | Depth cm | Dating years | Sample code | Depth cm | Dating years | Sample code | Depth cm | Dating years |
|---|---|---|---|---|---|---|---|---|
| C2-1 | 1 | 2007 | C1-1e2 | 1 e 2 | 2006 | C3-1e2 | 1 e 2 | 2003 |
| C2-2 | 2 | 2005 | C1-3 | 3 | 2003 | C3-3 | 3 | 2000 |
| C2-3 | 3 | 2004 | C1-4 | 4 | 2001 | C3-4 | 4 | 1997 |
| C2-4 | 4 | 2003 | C1-5 | 5 | 1999 | C3-5 | 5 | 1994 |
| C2-5 | 5 | 2001 | C1-6 | 6 | 1997 | C3-6 | 6 | 1991 |
| C2-6 | 6 | 2000 | C1-7 | 7 | 1996 | C3-7 | 7 | 1988 |
| C2-7 | 7 | 1999 | C1-8 | 8 | 1994 | C3-8 | 8 | 1985 |
| C2-8 | 8 | 1997 | C1-9 | 9 | 1992 | C3-9 | 9 | 1982 |
| C2-9 | 9 | 1996 | C1-10 | 10 | 1990 | C3-10 | 10 | 1979 |
| C2-10 | 10 | 1994 | C1-11 | 11 | 1989 | C3-11 | 11 | 1976 |
| C2-11 | 11 | 1993 | C1-12 | 12 | 1987 | C3-12 | 12 | 1973 |
| C2-12 | 12 | 1992 | C1-13 | 13 | 1985 | C3-13 | 13 | 1970 |
| C2-13 | 13 | 1990 | C1-14 | 14 | 1984 | C3-14 | 14 | 1967 |
| C2-14 | 14 | 1989 | C1-15 | 15 | 1982 | C3-15 | 15 | 1964 |
| C2-15 | 15 | 1988 | C1-16 | 16 | 1980 | C3-16 | 16 | 1961 |
| C2-16 | 16 | 1986 | C1-17 | 17 | 1978 | C3-17 | 17 | 1958 |
| C2-17 | 17 | 1985 | C1-18 | 18 | 1977 | C3-18 | 18 | 1955 |
| C2-18 | 18 | 1984 | C1-19 | 19 | 1975 | C3-19 | 19 | 1952 |
| C2-19 | 19 | 1982 | C1-20 | 20 | 1973 | C3-20 | 20 | 1949 |
| C2-20 | 20 | 1981 | C1-21 | 21 | 1972 | C3-21 | 21 | 1946 |
| C2-21 | 21 | 1979 | C1-22 | 22 | 1970 | C3-22 | 22 | 1943 |
| C2-22 | 22 | 1978 | C1-23 | 23 | 1968 | C3-23 | 23 | 1940 |
| C2-23 | 23 | 1977 | C1-24 | 24 | 1966 | C3-24 | 24 | 1937 |
| C2-24 | 24 | 1975 | C1-25 | 25 | 1964 | C3-25 | 25 | 1934 |
| C2-25 | 25 | 1974 | C1-26 | 26 | 1962 | C3-26 | 26 | 1931 |
| C2-26 | 26 | 1973 | C1-27 | 27 | 1960 | C3-27 | 27 | 1928 |
| C2-27 | 27 | 1971 | C1-28 | 28 | 1957 | C3-28 | 28 | 1925 |
| C2-28 | 28 | 1970 | C1-29 | 29 | 1956 | C3-29 | 29 | 1922 |
| C2-29 | 29 | 1968 | C1-30 | 30 | 1955 | C3-30 | 30 | 1919 |
| C2-30 | 30 | 1967 | C1-32 | 32 | 1951 | C3-32 | 32 | 1915 |
| C2-32 | 32 | 1965 | C1-34 | 34 | 1948 | C3-34 | 34 | 1909 |
| C2-34 | 34 | 1962 | C1-36 | 36 | 1944 | C3-36 | 36 | 1903 |
| C2-36 | 36 | 1960 | C1-38 | 38 | 1941 | C3-38 | 38 | 1897 |
| C2-38 | 38 | 1957 | C1-40 | 40 | 1937 | C3-40 | 40 | 1891 |
| C2-40 | 40 | 1954 | C1-42 | 42 | 1934 | C3-42 | 42 | 1885 |
| C2-42 | 42 | 1951 | C1-44 | 44 | 1930 | C3-44 | 44 | 1879 |
| C2-44 | 44 | 1949 | C1-46 | 46 | 1927 | C3-46 | 46 | 1873 |
| C2-46 | 46 | 1946 | C1-48 | 48 | 1924 | C3-48 | 48 | 1867 |
| C2-48 | 48 | 1943 | C1-50 | 50 | 1921 | C3-50 | 50 | 1861 |
| C2-50 | 50 | 1940 | C1-52 | 52 | 1918 | | | |
| C2-52 | 52 | 1938 | C1-54 | 54 | 1915 | | | |
| C2-54 | 54 | 1935 | C1-56 | 56 | 1912 | | | |
| C2-56 | 56 | 1932 | C1-58 | 58 | 1909 | | | |
| C2-58 | 58 | 1929 | C1-60 | 60 | 1906 | | | |
| C2-60 | 60 | 1927 | C1-62 | 62 | 1903 | | | |
| C2-62 | 62 | 1924 | C1-64 | 64 | 1900 | | | |
| C2-66 | 66 | 1918 | C1-66 | 66 | 1897 | | | |
| C2-68 | 68 | 1916 | C1-68 | 68 | 1895 | | | |
| C2-70 | 70 | 1913 | C1-70 | 70 | 1892 | | | |
| C2-72 | 72 | 1910 | C1-72 | 72 | 1889 | | | |
| C2-74 | 74 | 1908 | C1-74 | 74 | 1887 | | | |
| C2-76 | 76 | 1905 | C1-76 | 76 | 1884 | | | |
| C2-78 | 78 | 1902 | C1-78 | 78 | 1881 | | | |
| C2-80 | 80 | 1899 | C1-80 | 80 | 1879 | | | |
| C2-82 | 82 | 1897 | C1-82 | 82 | 1876 | | | |
| C2-84 | 84 | 1894 | C1-84 | 84 | 1874 | | | |
| C2-86 | 86 | 1891 | C1-86 | 86 | 1871 | | | |
| C2-88 | 88 | 1888 | C1-88 | 88 | 1868 | | | |
| C2-90 | 90 | 1886 | C1-90 | 90 | 1865 | | | |
| C2-92 | 92 | 1883 | C1-92 | 92 | 1863 | | | |
| C2-94 | 94 | 1880 | C1-95 | 95 | 1859 | | | |
| C2-96 | 96 | 1877 | | | | | | |
| C2-98 | 98 | 1875 | | | | | | |
| C2-100 | 100 | 1872 | | | | | | |
| C2-102 | 102 | 1869 | | | | | | |

Fig. SM2: The table shows the sub-sample lists for all 3 sediment cores. Each sample is associated with: an alphanumeric code, depth to surface and corresponding date.

Table SM1: Measurement wavelengths by ICP-OES.

| Element | $\lambda$ (nm) |
|---|---|
| Zn | 213,856 |
| P | 214,914 |
| Ni | 216,556 |
| Pb | 220,353 |
| Co | 228,616 |
| Al | 237,312 |
| Mn | 257,61 |
| Fe | 259,94 |
| V | 292,402 |
| Cr | 267,716 |
| Ti | 323,452 |
| Cu | 327,396 |

Table SM2: LOD values per element are reported.

| Element | LOD | | |
|---|---|---|---|
| | C1 | C2 | C3 |
| Mg | 2,69 | 1,07 | 2,69 |
| Fe | 0,12 | 0,22 | 0,12 |
| Al | 1,01 | 0,12 | 1,01 |
| Ca | 2,95 | 0,56 | 2,95 |
| K | 2,63 | 0,32 | 2,63 |
| V | 0,09 | 0,02 | 0,26 |
| Co | 0,12 | 0,02 | 0,20 |
| Ni | 0,09 | 0,03 | 0,10 |
| Pb | 0,07 | 0,02 | 0,19 |
| Cr | 0,06 | 0,01 | 0,20 |
| Cu | 0,04 | 0,01 | 0,37 |
| Ti | 0,01 | 0,04 | 0,28 |
| Zn | 0,03 | 0,03 | 0,08 |
| P | 0,03 | 0.03 | 0,15 |
| Mn | 0,01 | 0,16 | 0,05 |

# References

Bazzano A, Grotti M (2014) Determination of lead isotope ratios in environmental matrices by quadrupole ICP-MS working at low sample consumption rates. Journal of Analytical Atomic Spectrometry 29(5):926. doi:https://doi.org/10.1039/c3ja50388g

Bazzano A, Bertinetti S, Ardini F, et al (2021) Potential Source Areas for Atmospheric Lead Reaching Ny-Ålesund from 2010 to 2018. Atmosphere

| | Element | **Mean Bottom below 76 cm** | dev sta | **Mean upper 76 cm** | dev sta | **Mean whole core** | dev sta |
|---|---|---|---|---|---|---|---|
| **C1** | Co | 10,7 | 3,7 | 13,1 | 2,2 | 12,7 | 2,6 |
| | Ni | 109.0 | 10.9 | 99.1 | 12.8 | 100.7 | 13,0 |
| | P | 1497,8 | 77,3 | 1723,6 | 304,7 | 1686,6 | 292,2 |
| | Pb | 13,5 | 2,6 | 18,6 | 2,8 | 17,8 | 3,3 |
| | Cr | 144,1 | 10,2 | 131,3 | 13,7 | 133,4 | 14,0 |
| | Cu | 24,4 | 8,4 | 32,3 | 5,7 | 31,0 | 6,8 |
| | Ti | 406,8 | 65,2 | 317,4 | 70,3 | 332,0 | 76,6 |
| | Zn | 87,2 | 19,4 | 97,8 | 28,6 | 96,0 | 27,5 |
| | Mn | 781,0 | 64,4 | 829,6 | 105,5 | 821,7 | 101,1 |
| | V | 73,2 | 8,6 | 77,0 | 11,5 | 76,4 | 11,1 |
| **C2** | Co | 20,5 | 1,5 | 19,6 | 4,4 | 19,8 | 4,0 |
| | Ni | 117,4 | 6,7 | 101,3 | 25,1 | 104,8 | 23,3 |
| | P | 2310,9 | 119,9 | 2448,7 | 223,8 | 2419,0 | 212,8 |
| | Pb | 19,6 | 1,3 | 22,6 | 2,8 | 22,0 | 2,8 |
| | Cr | 183,4 | 17,2 | 158,8 | 39,8 | 164,1 | 37,5 |
| | Cu | 25,2 | 1,9 | 24,2 | 2,7 | 24,4 | 2,6 |
| | Ti | 514,4 | 118,0 | 446,9 | 121,2 | 461,8 | 122,8 |
| | Zn | 115,2 | 8,5 | 111,9 | 21,6 | 112,6 | 19,5 |
| | Mn | 815,7 | 117,6 | 1312,5 | 388,9 | 1205,5 | 404,1 |
| | V | 98,3 | 11,6 | 93,2 | 15,8 | 94,3 | 15,1 |
| **C3** | Co | 4,9 | 3,5 | 8,8 | 4,0 | 7,9 | 4,2 |
| | Ni | 71,9 | 6,6 | 80,8 | 11,0 | 78,6 | 10,7 |
| | P | 1277,8 | 183,8 | 1323,3 | 155,6 | 1311,2 | 161,9 |
| | Pb | 9,4 | 1,9 | 14,2 | 2,3 | 13,0 | 3,0 |
| | Cr | 90,8 | 13,9 | 111,6 | 19,7 | 106,4 | 20,4 |
| | Cu | 13,0 | 2,0 | 19,6 | 3,0 | 18,0 | 4,0 |
| | Ti | 307,2 | 63,7 | 356,2 | 121,8 | 343,3 | 110,7 |
| | Zn | 58,6 | 9,2 | 86,9 | 37,3 | 79,8 | 34,8 |
| | Mn | 709,6 | 58,4 | 730,8 | 111,0 | 725,5 | 100,1 |
| | V | 47,1 | 7,9 | 69,7 | 13,4 | 64,1 | 15,7 |
| **Current State** | Co | 7,7 | 3,7 | | | | |
| | Ni | 76,6 | 16,2 | | | | |
| | P | 418,6 | 79,1 | | | | |
| | Pb | 18,8 | 3,0 | | | | |
| | Cr | 100,0 | 27,6 | | | | |
| | Cu | 18,2 | 4,1 | | | | |
| | Ti | 292,5 | 104,4 | | | | |
| | Zn | 78,5 | 13,1 | | | | |
| | Mn | 1063,9 | 303,9 | | | | |
| | V | 53,3 | 17,6 | | | | |

Table SM3: Average, maximum and minimum concentrations (unit mg/Kg) for the whole sediment cores (C1,C2,C3), average of the anthropized (upper) and average of the pristine (bottom) section are reported.

12(3):388. doi:https://doi.org/10.3390/atmos12030388

Bertinetti S, Bolea-Fernandez E, Malandrino M, et al (2022) Strontium isotopic analysis of environmental microsamples by inductively coupled plasma – tandem mass spectrometry. Journal of Analytical Atomic Spectrometry 37(1):103–113. doi:https://doi.org/10.1039/D1JA00329A

Table SM4: Principal component analysis on C2 core, by euclidean distance and complete method. The principal component values major are highlighted in each column.

| Element | Component | | |
|---|---|---|---|
| | **PC1** | **PC2** | **PC3** |
| Mn | -0.276 | 0.203 | 0.351 |
| Fe | 0.192 | 0.106 | 0.498 |
| Al | 0.246 | -0.366 | 0.384 |
| V | 0.357 | -0.164 | 0.150 |
| Co | 0.356 | 0.238 | -0.032 |
| Ni | 0.351 | 0.200 | -0.166 |
| Pb | 0.153 | 0.503 | 0.310 |
| Cr | 0.378 | 0.018 | -0.030 |
| Cu | 0.014 | -0.405 | -0.075 |
| Ti | 0.248 | -0.419 | 0.191 |
| Zn | 0.355 | 0.194 | -0.011 |
| P | -0.168 | -0.175 | 0.435 |
| PAHs | -0.256 | 0.169 | 0.318 |
| Proportion of Variance | 0.522 | 0.146 | 0.128 |
| Cumulative Proportion | 0.522 | 0.667 | 0.796 |

Marchegiano M, Francke A, Gliozzi E, et al (2018) Arid and humid phases in central Italy during the Late Pleistocene revealed by the Lake Trasimeno ostracod record. Palaeogeography, Palaeoclimatology, Palaeoecology 490:55–69. doi:https://doi.org/10.1016/j.palaeo.2017.09.033

Vanhaecke F, Balcaen L, Malinovsky D (2009) Use of single-collector and multi-collector ICP-mass spectrometry for isotopic analysis. Journal of Analytical Atomic Spectrometry 24(7):863. doi:https://doi.org/10.1039/b903887f

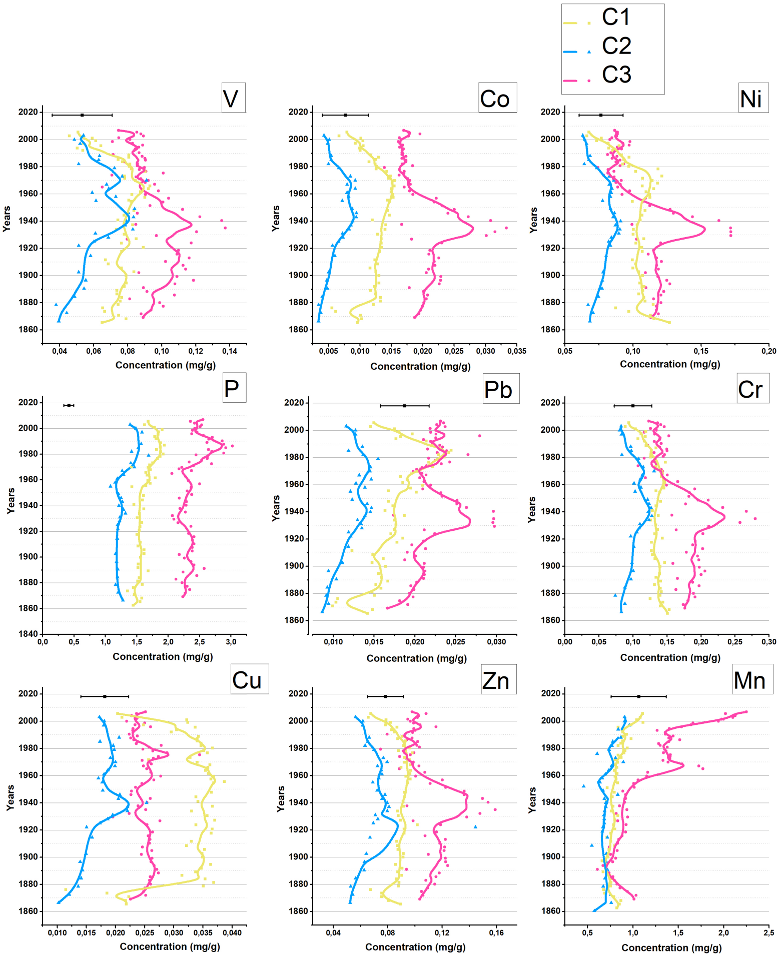


Fig. SM3: Trace element concentration trends (V, Co, Ni, P, Pb, Cr, Cu, Zn and Mn) in the three sediment cores: C1 (yellow), C2 (pink), C3 (blue) (adjacent-averaging method, with weighted average options was used on 5 points to obtain the trends line). The scatter represent single samples analysed and the line represents moving averages every 5 years. The black square represents the current concentration in the surface sediments (corresponding to 2018) and the error bar represents standard deviation.

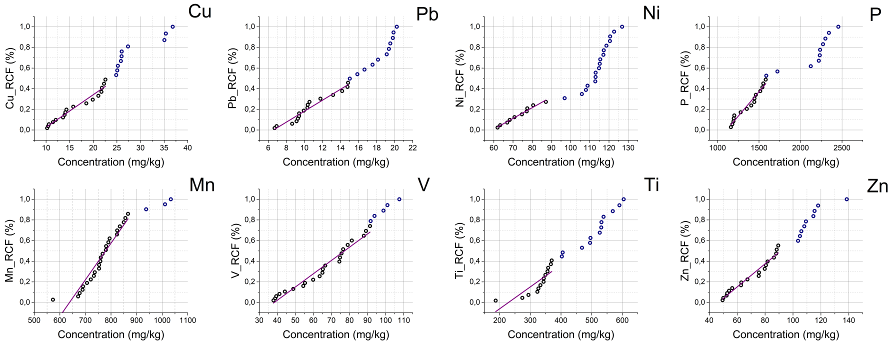


Fig. SM4: Cumulative frequency curves (scatters) of Cu, Pb,Ni, P, Mn, V, Ti and Zn. Linear regressions were performed on the cumulative frequency curve with $p<0.01$ and $R2>0.9$ (purple line).

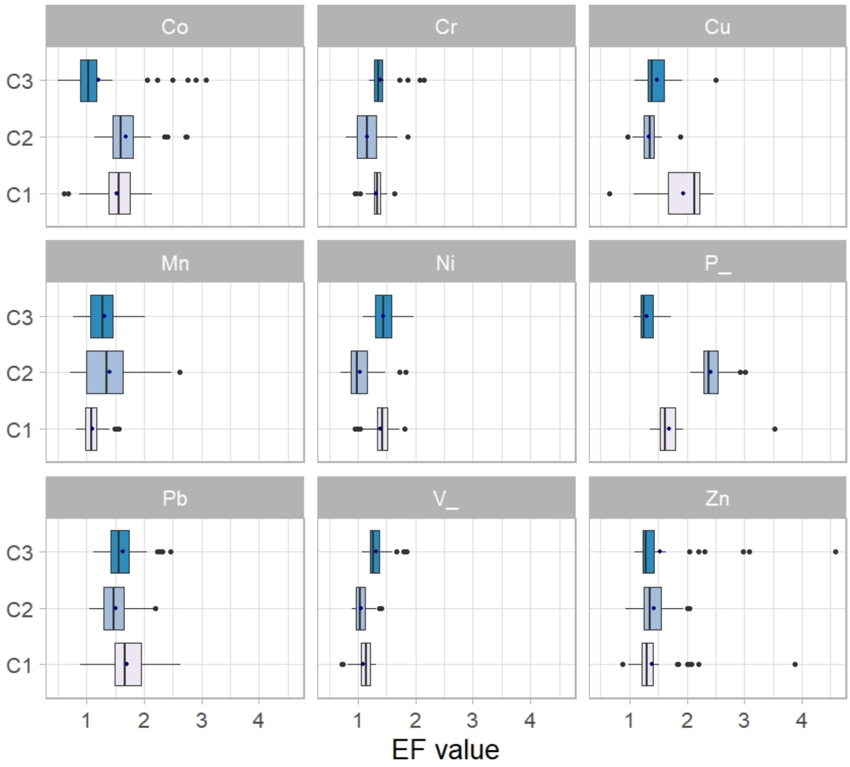


Fig. SM5: Boxplot of Enrichment factor for trace elements calculated for the 3 sediment cores. The mean values are represented by blue point.

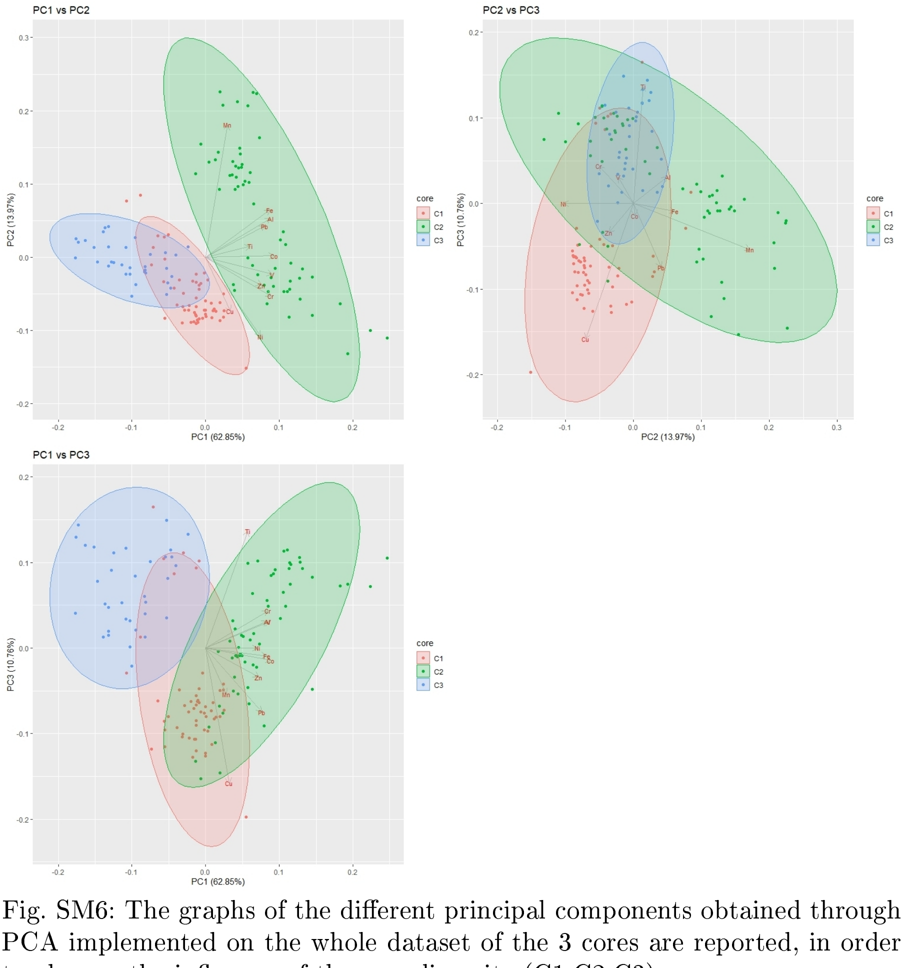


Fig. SM6: The graphs of the different principal components obtained through PCA implemented on the whole dataset of the 3 cores are reported, in order to observe the influence of the sampling site (C1,C2,C3).

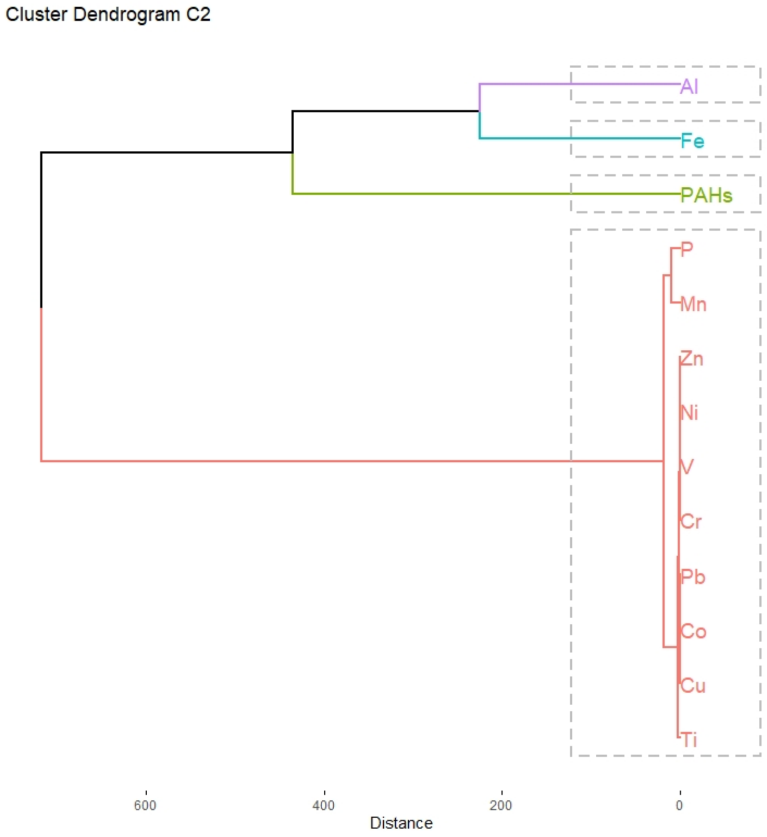


Fig. SM7: Hierarchical graph obtained with the cluster analysis for core C2 is shown. Al and Fe are grouped by the purple and blue boxes, PAHs by the green box, the other anthropogenic elements are grouped by the pink box, but Mn and P are indicated by a separate subgroup.

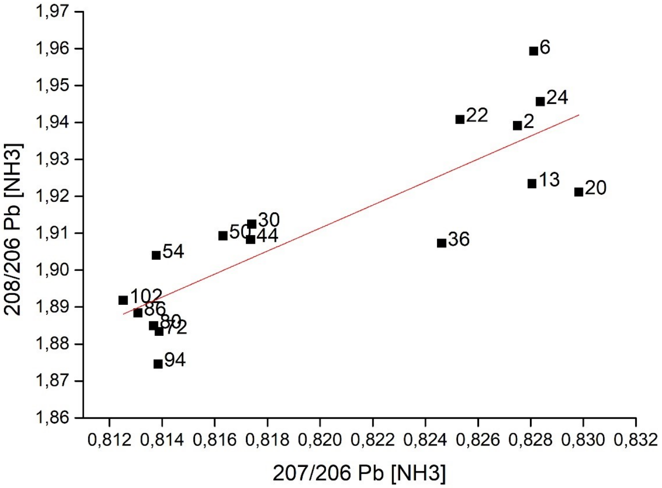


Fig. SM8: $^{208}/^{206}$Pb vs. $^{207}/^{206}$Pb; the labels correspond to the sample number.
